# TRAF1 suppresses antifungal immunity through CXCL1-mediated neutrophil recruitment during *Candida albicans* intradermal infection

**DOI:** 10.1186/s12964-020-00532-x

**Published:** 2020-02-24

**Authors:** Wenjuan Bai, Qingqing Wang, Zihou Deng, Tiantian Li, Hui Xiao, Zhiyuan Wu

**Affiliations:** 1 Pediatric Intensive Care Unit, Guangzhou Women and Children’s Medical Center, |Guangzhou Medical University, 9 Jinsui Road, Guangzhou, Guangdong 510120 People’s Republic of China; 2 Key Laboratory of Molecular Virology and Immunology, Institut Pasteur of Shanghai, Chinese Academy of Sciences, Shanghai, 200031 People’s Republic of China

**Keywords:** CXCL1, TRAF1, *C. albicans*, Skin infection, Neutrophil

## Abstract

**Background:**

*Candida albicans* is the most common opportunistic human fungal pathogen. The chemokine ligand CXCL1 plays a protective role in fungal infection through the recruitment of neutrophils. TRAF1 (tumor necrosis factor-associated factor 1) can be highly induced by proinflammatory stimuli such as LPS and TNF and has been implicated in septic shock. However, the role of TRAF1 in infection, especially fungal infection, remains elusive. Herein, we reveal that TRAF1 suppresses the antifungal immune response to *Candida albicans* intradermal infection through the regulation of CXCL1 induction and neutrophil recruitment.

**Methods:**

A mouse model of *C. albicans* intradermal infection was established. The *Traf1*^−/−^ mice and *Traf1*^−/−^ immortalized human keratinocytes were generated. The p65 inhibitor triptolide, STAT1 inhibitor fludarabine, neutrophil-depletion antibody Ly6G, and neutralizing antibody for CXCL1 were utilized. The expression of proinflammatory cytokines and chemokines was assessed by real-time PCR and ELISA, and the activation of signaling molecules was analyzed by Western blotting. Hematoxylin and eosin staining and periodic acid Schiff staining were used for histology or fungal detection, respectively. The immunofluorescence and flow cytometry analyses were employed in the assessment of immune cell infiltration. Bone marrow transplantation and adoptive transfer experiments were conducted to establish a role for TRAF1 in the macrophage compartment in fungal skin infection.

**Results:**

TRAF1-deficient mice demonstrated improved control of *Candida albicans* intradermal infection, and concomitant increase in neutrophil recruitment and reduction in fungal burden. The chemokine CXCL1 was upregulated in the TRAF1-deficient macrophages treated with heat-killed *C. albicans*. Mechanistically, TRAF1-deficient macrophages showed increased activation of transcription factor NFκB p65. The human CXCL8 was also highly induced in the TRAF1-deficient human keratinocytes upon TNF stimulation through decreasing the activation of transcription factor STAT1. TRAF1-deficient macrophages played a critical role in containing the *C. albicans* skin infection in vivo.

**Conclusion:**

TRAF1-deficient mice can better control fungal infection in the skin, a process attributable to the CXCL-neutrophil axis. Mechanistically, TRAF1 likely regulates CXCL1 expression in both macrophages and keratinocytes through the transcriptional factor NFκB and STAT1, respectively. Our finding offers new insight into the understanding of the immune regulatory mechanisms in host defense against *C. albicans* infection.

**Graphical abstract:**

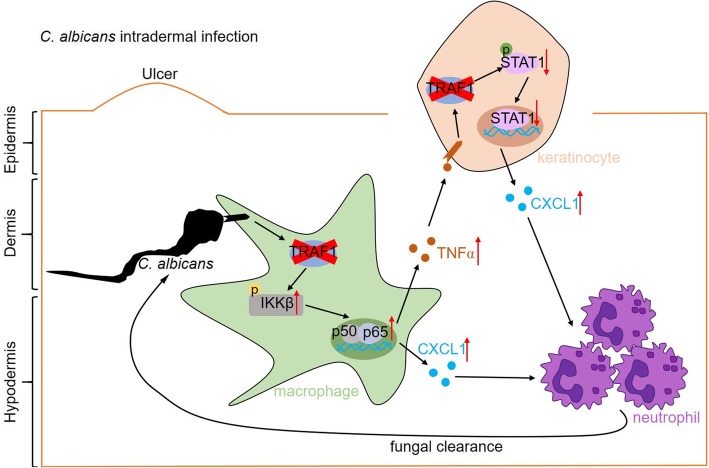

## Highlights

- TRAF1-deficiency protects mice from the intradermal infection with *Candida albicans*.
- CXCL1-upregulated by TRAF1-deficiency mediates the recruitment of neutrophils for fungal clearance.
- TRAF1-deficiency leads to increased NFκB activation in the macrophages.
- TRAF1-deficiency ablates STAT1 activation in the keratinocytes.

## Background

*Candida albicans* is the most common human opportunistic fungal pathogen, which commensally localizes on the skin and mucous surface of healthy people. Patients with diabetes mellitus, Acquired Immune Deficiency Syndrome (AIDS), chronic systemic corticosteroid usage, chemotherapy-induced neutropenia, or IL-23/ IL-17A blockade for the treatment of autoimmune diseases such as rheumatoid arthritis, as well as patients at ICU or with impaired immunity are predisposed to chronic mucosal and cutaneous candidiasis (CMC) or even systemic candidiasis, leading to significant morbidity and greater than 50% mortality [1–9]. The current treatment for fungal infection is very limited, and there is widespread resistance for the anti-fungal drugs. However, we have very limited understanding of the immune mechanisms required for anti-fungal defense, which severely hinders the development of effective therapeutic approaches to contain the fungal infection. Humans with inherited deficiency of CARD9 (caspase recruitment domain) are susceptible to fungal infection in the CNS (central nervous system) [10], suggesting the involvement of dectin-1 signaling in anti-fungal infection.

Immune-related genes complement component 5 (C5)/TRAF1 located on Chromosome 9q33–34 is identified as a risk factor for rheumatoid arthritis [11], uveitis in juvenile idiopathic arthritis [12], multiple autoimmune diseases such as SLE [13]. TRAF1 is associated with susceptibility to autoimmune thyroid disease [14], IBD [15] and DMBA/solar UVR-induced skin carcinogenesis [16]. However, the role of TRAF1 in infectious diseases such as *C. albicans* infection remains unknown. During *C. albicans* skin infection, CD301b^+^ dermal dendritic cells (dDC) release IL-23, which acts on dermal gamma delta T lymphocyte cells to produce IL-17. Subsequently, IL-17 induces the expression of CXCL1 and G-CSF, leading to the recruitment and activation of neutrophils. Neutrophils and macrophages are the principal innate immune cells required for the phagocytosis and killing of *C. albicans* [17].

TRAF1 was firstly discovered as an adaptor of the TNFR2 (Tumor necrosis factor receptor 2) signaling complex and TRAF1 negatively regulates TNFR2 signaling [18]. TRAF1 is a unique member of the TRAF family due to the lacking of the RING finger domain, and thus the E3 ubiquitin ligase activity. TRAF1 is constitutively expressed in only limited tissues such as skin, spleen, lung, and testis, implicating its unique function in these tissues. TRAF1 can inhibit the linear ubiquitination of NEMO by binding the three components of the linear ubiquitin assembly complex (LUBAC), thereby downregulating the activation of NF-κB (nuclear factor-kappa B) [19]. Accordingly, TRAF1 plays a negative role in LPS (lipopolysaccharide)-TLR4-mediated inflammatory response. Nevertheless, the role of TRAF1 in the regulation of *C. albicans*-induced inflammatory signaling remains unknown.

In the present study, we established a mouse model of *C. albicans* intradermal infection and investigated the role of TRAF1 in antifungal immune response. Our results indicate that *C. albicans*-elicited ulceration and tissue damage were ameliorated in TRAF1-deficient mice. On the other hand, we observed increased expression of chemokines such as CXCL1 and prominent recruitment of neutrophils in *Traf1*^−/−^ skin following *C. albicans* infection. Further, TRAF1-deficiency led to increased expression of CXCL1 in the macrophages treated with heat-killed *C. albicans*, likely attributing to elevated activation of NFκB p65. Moreover, TRAF1-deficiency resulted in a lower activation of STAT1 and more expression of CXCL8 in the human immortalized keratinocytes in response to TNFα stimulation. Importantly, neutralization of CXCL1 or depletion of neutrophils compromised the immune defense mechanisms against *C. albicans* in the TRAF1-deficient mice. Collectively, our data unveil TRAF1 as a critical regulator of the immune defense against *C. albicans* intradermal infection.

## Materials and methods

### Mice

The *Traf1*^−/−^ mice (C.129S4-Traf1tm1Tsi/TsiPryhJ) were purchased from Jackson Laboratories (Bar Harbor, ME) and then backcrossed onto the C57BL/6 background for eight generations. The *Rag1*^*−/−*^ mice (002216- B6.129S7-Rag1^tm1Mom^/J, Jackson Laboratories) were bred with *Traf1*^*−/−*^ mice to generate *Rag1*^*−/−*^*Traf1*^*−/−*^ mice. All the mice were housed in sterile microisolator cages under the specific pathogen-free conditions at Institute Pasteur of Shanghai. The sex- and age- matched female littermates at 6–12 weeks of age were used for all the experiments. The animal studies were conducted in compliance with a protocol (No. P2019036) approved by the Institutional Animal Care and Use Committee at Institut Pasteur of Shanghai.

### *C. albicans* culture and heat-inactivation

A single colony of *C. albicans* SC5314 was inoculated into the yeast peptone dextrose medium and cultured O/N at 30 °C. The Fungal cells from late stationary phase culture were transferred to fresh yeast peptone dextrose medium (1:100 dilution), incubated at 30 °C until mid-exponential phase. The fungal culture was then collected by centrifugation (7000 rpm, 1 min), and counted for infection. The fungi resuspended in phosphate buffer saline were inactivated at 65 °C for 2 h.

### *C. albicans* intradermal infection model

Mice at 8 to 12 weeks of age were shaved one day prior to infection, allowing better visualization on the infection site and accurate measurement of the abscess. All mouse procedures were performed under general anesthesia by intraperitoneal injection of 70 μg of pentobarbital sodium/gram mouse weight. Animals were intradermally injected with 0.1 mL of 1 × 10 ^7^ live *C. albicans* or sterile phosphate buffer saline. The ulcer was measured by length (L) and width (W) with the caliper at 1, 3, 5, 7 days post-infection. The ulcer length and width dimensions were used to calculate the ulcer area: π×(L/2) × (W/2). An objective scoring system was developed to evaluate the severity of skin infection, based on the severity of ulceration, scab, erythema, and nodule, which were scored independently on a scale from 0 to 4: 0, none; 1, slight; 2, moderate; 3, marked; 4, very marked. The cumulative score served as a measure of the severity of the infected skin (scale 0–16), which was scored at 1, 3, 5, 7 days post-infection. The observer was blinded to all biopsy specimens.

### Histological analysis and PAS staining

After the mice were killed 3 days post-infection, 7 mm diameter-biopsy specimens of skin were immediately excised and immediately fixed in phosphate-buffered (pH 7.4) formalin (4%). The formalin-fixed biopsy specimens were embedded in paraffin and stained by H&E (hematoxylin and eosin) for histological analysis and stained by PAS (periodic acid Schiff) for the detection of fungal pathogens. Otherwise, the formalin-fixed biopsy specimens were embedded in OCT (optimum cutting temperature) compound and stained by PAS for fungi.

### Fungal burden determination

The mice were injected with 1 × 10^7^ CFU (colony-forming units) *C. albicans*. On day 3 post-infection, the mice were sacrificed and the skin tissues were homogenized in PBS and serially diluted before plating on to yeast peptone dextrose agar plates supplemented with penicillin/streptomycin (Invitrogen). The colonies were counted after incubation for 36 h at 30 °C.

### Immunofluorescence

Skin tissues from *C. albicans*- infected mice for 3 days were harvested and immediately fixed in phosphate-buffered (pH 7.4) formalin (4%). The formalin-fixed biopsy specimens were embedded in OCT medium at − 20 °C. The tissues were then cut into 5 μm sections by a cryostat and mounted on glass slides, which were then dried for at least 1 h before being stored at − 20 °C. The tissue sections were fixed and permeabilized with ice-cold acetone, followed by blocking with 1% BSA for 1 h. Ly6G (RB6-8C5; BD) mAb was used as the first antibody, and the goat-anti-Rat-CY3 (InvivoGen) mAb was used as the secondary antibody and DAPI was used to stain the nucleus. The stained tissues were mounted via a prolong gold antifade mounting (Beyotime), and the images were captured using a fluorescence microscope (Olympus IX73). The positive cells were counted in 5 fields per tissue section and the mean value represented the infiltrating neutrophils of each mouse.

### Depletion and neutralization of neutrophils

Mice were treated with 40 μg of Ly6G mAb (1A8, BD) or rat IgG control (R&D Systems) through intraperitoneal injection 2 h before infection. The skin tissues from the day 3 post-infected mice were harvested and frozen in OCT medium for the immunofluorescence and PAS staining. The mice were also treated with 40 μg of anti-CXCL1 mAb (R&D Systems) or rat IgG control (R&D Systems) via intraperitoneal injection 2 h prior to infection. Mice were sacrificed one day after infection and skin tissues were frozen in OCT medium and stained by immunofluorescence and PAS, respectively.

### Isolation of single cells from skin tissue

Mouse dorsal uninfected or infected skin was harvested, washed with PBS and then cut into 1–2 mm^2^ pieces, and digested with 1 mg/ml collagenase IV (Invitrogen) in RPMI 1640 medium containing 10% FBS at 37 °C for 1 h with shaking. Digested cells were grinded and passed through 40-μm cell strainers (BD Biosciences). Then immune cells were enriched by percoll centrifugation according to manufacturer’s instructions (GE).

### Flow Cytometry

Fluorochrome-labeled antibodies for Fixable Viability Dye, CD45.2, CD11b, Ly6G, Ly6C, F4/80, B220 were from eBioscience. Antibody for CD3 was from BD Biosciences. For cell surface marker staining, skin single cells and bone marrow cells were incubated with specific antibodies for 30 mins on ice, followed by washing with MACS buffer (PBS, 2% FBS, 2.5 mM EDTA) twice. Subsequently, cells were acquired using LSR-Fortessa flow cytometer (BD), and the data were analyzed by Flow Jo V10 software.

### Generation of bone marrow-derived macrophages

Bone marrow cells were isolated by flushing femurs and tibia of 6–8 weeks mice with RPMI 1640 medium (Invitrogen). Red blood cells were lysed using ACK lysis buffer (0.15 M NH_4_Cl, 1 mM KHCO_3_, 0.1 mM Na_2_EDTA, pH 7.3). To generate BMDM (bone marrow-derived macrophages), bone marrow cells were cultured in RPMI 1640 medium supplemented with 30% L929-conditioned medium (containing about 20 ng/ml M-CSF), as well as 10% FBS. On day 4, the non-adherent cells were removed and fresh RPMI with L929-conditioned medium was added. The BMDMs were used on day 7.

### Generation of TRAF1 deficiency in immortalized human keratinocytes

Guide RNAs targeting *Traf1* gene were designed using the online optimized software (http://crispr.mit.edu). Three guide RNAs were inserted into LentiCRISPR (pXPR_001) vector. The recombinant and empty plasmids were transiently transfected into 293 T cells, and the supernatants were collected after 48 h. Immortalized human keratinocytes (HaCaT cells) were infected by packaged Lentivirus with puromycin to screen positive clones, which were further identified by immunoblot analysis and the sg-*Traf1* pool with guide RNA oligo sequences F: 5′- CACCGAGGAAGCCGTCTTCGAAC -3′, R: 5′- AAACGAGTTCGAAGACGGCTTCCTC − 3′ to knockout TRAF1 was used in this study.

### RNA preparation and real-time PCR

RNAs were extracted from the skin tissues with or without *C. albicans* infection for 3 days, BMDMs stimulated by heat-killed *C. albicans* (HK-CA) for 6 h with or without triptolide (20 ng/ml, Selleck) pretreatment for 2 h, or HaCaT cells stimulated with TNFα (20 μg/ml) plus IL-17A (50 μg/ml) for 9 h with or without fludarabine (100 μM, Selleck; 4 h) by TRIZOL (Invitrogen) according to the manufacture’s instruction. The cDNAs were reversely transcribed from 0.5 μg total RNA by PrimeScript™ RT-PCR Kit (Takara). The real-time PCR (qPCR) was carried out with PrimeScript® RT reagent Kit (Takara) on ABI 7900HT Fast qPCR System. The relative expression of target genes was presented as fold change normalized to the expression of β-actin and relative to the untreated control by using ΔΔct method. All the qPCR samples were analyzed in triplicate in each experiment, and each experiment was replicated at least three times. All the qPCR primers used in this study are described in Table 1.

**Table 1:** List of primers used for qPCR

Gene	Forward primer	Reverse primer
m-*Cxcl1*	GCTGGGATTCACCTCAAGAA	CTTGGGGACACCTTTTAGCA
m-*Cxcl2*	GCCAAGGGTTGACTTCAAGAAC	GCTTCAGGGTCAAGGCAAACT
m-*Il6*	AGATAAGCTGGAGTCACAGAAGGAG	CGCACTAGGTTTGCCGAGTAG
m-*Il23α*	CACCAGCGGGACATATGAATCTA	CAGAACTGGCTGTTGTCCTTGA
m-*Tnfα*	GTCCCCAAAGGGATGAGAAGTT	GTTTGCTACGACGTGGGCTACA
m-*iNOS*	GGCAGCCTGTGAGACCTTTG	CATTGGAAGTGAAGCGTTTCG
m-*Il1β*	CAACCAACAAGTGATATTCTCCATG	GATCCACACTCTCCAGCTGCA
m-*β-actin*	CCAGCCTTCCTTCTTGGGTAT	AGAGGTCTTTACGGATGTCAACG
h-*Cxcl8*	GCAGCTCTGTGTGAAGGTGC	TCTGCACCCAGTTTTCCTTG
h-*S100A9*	CAAAGAGCTGGTGCGAAAAG	CGAAGCTCAGCTTGTCT
h-*BD2*	GCCATCAGCCATGAGGGTCTTG	AATCCGCATCAGCCACAGCAG
h-*Il1β*	CCTTGGGCCTCAAGGAAAA	CTCCAGCTGTAGAGTGGGCTTA
h-*β-actin*	TACGCCAACACAGTGCTGTCT	TCTGCATCCTGTCGGCAA

m: *Mus musculus*; h: *Homo sapiens*

### Elisa

Secreted CXCL1 was measured according to the manufacturers’ protocol (Peprotech) in the culture supernatants of BMDM stimulated with or without HK-CA for 24 h.

### Western blotting analysis

The whole-cell lysates were suspended in lysis buffer containing protease inhibitor (complete mini, Roche), 1 mM PMSF, 1 mM Na_3_VO_4_ and 1 mM NaF. To attain cytosolic extract and nuclear extract, cells were lysed in hypotonic buffer (10 mM HEPES, PH 7.6, 1.5 mM MgCl_2_, 10 mM KCl, 1 mM EDTA, supplemented with protease inhibitor, 1 mM PMSF, 1 mM Na_3_VO_4_ and 1 mM NaF). Following centrifugation for 5 mins at 3000 rpm, supernatants were continued to centrifugate for 15 mins at 12000 rpm and then supernatants were collected as cytosolic extract; Nuclei-containing pellets were washed 3 times with hypotonic buffer and were lysed in high salt buffer (20 mM HEPES, PH 7.6, 500 mM NaCl, 1.5 mM MgCl_2_, 1 mM EDTA, supplemented with protease inhibitor, 1 mM PMSF, 1 mM Na_3_VO_4_ and 1 mM NaF). Following centrifugation for 15 mins at 12000 rpm, supernatants were collected as a nuclear extract. Lysates were separated in SDS-PAGE and transferred to polyvinylidene fluoride membrane (Bio-Rad Laboratories). The membrane was incubated with the following primary antibodies: p-IKKα/β, p-IκBα, p65, p50, p-STAT1, STAT1, TRAF1, β-actin, Histone H3 and GAPDH (Cell Signaling Technology) with a dilution of 1:1000.

### Bone marrow chimeric mice generation

Recipient mice (6 weeks old) were fed with enrofloxacin solution (Bayer, 800 μl enrofloxacin solution /L water) in drinking water for 2 weeks. Food but not water was removed one day prior to irradiation. Wipe down each container with 70% ethyl alcohol and put 4 mice into each clean container. Mice in container were exposed to 8 Gy total body X-ray irradiation administered in one dose and received bone marrow cells from donors (1 × 10^6^ cells suspended in 200 μl PBS /mouse) intravenously in 4 h. Subsequently, food was restored and enrofloxacin containing water were fed for 2 weeks. Then the regular water was restored for another 2 weeks.

### Macrophage adoptive transfer

BMDMs were resuspended in sterile PBS at a concentration of 10^7^ cells/ml and injected into the recipient mice intravenously, with 10^6^ macrophages in a total volume of 100 ul 4 h prior to *C. albicans* infection.

### Statistics

All the data are presented as mean ± SEM. The statistical analyses were analyzed with the two-tailed, unpaired, Student’s t-test, unless specified. In all cases, values of p below 0.05 considered statistically significant.

## Results

### TRAF1-deficiency protects mice from the intradermal infection with *C. albicans*

**Fig. 1:**
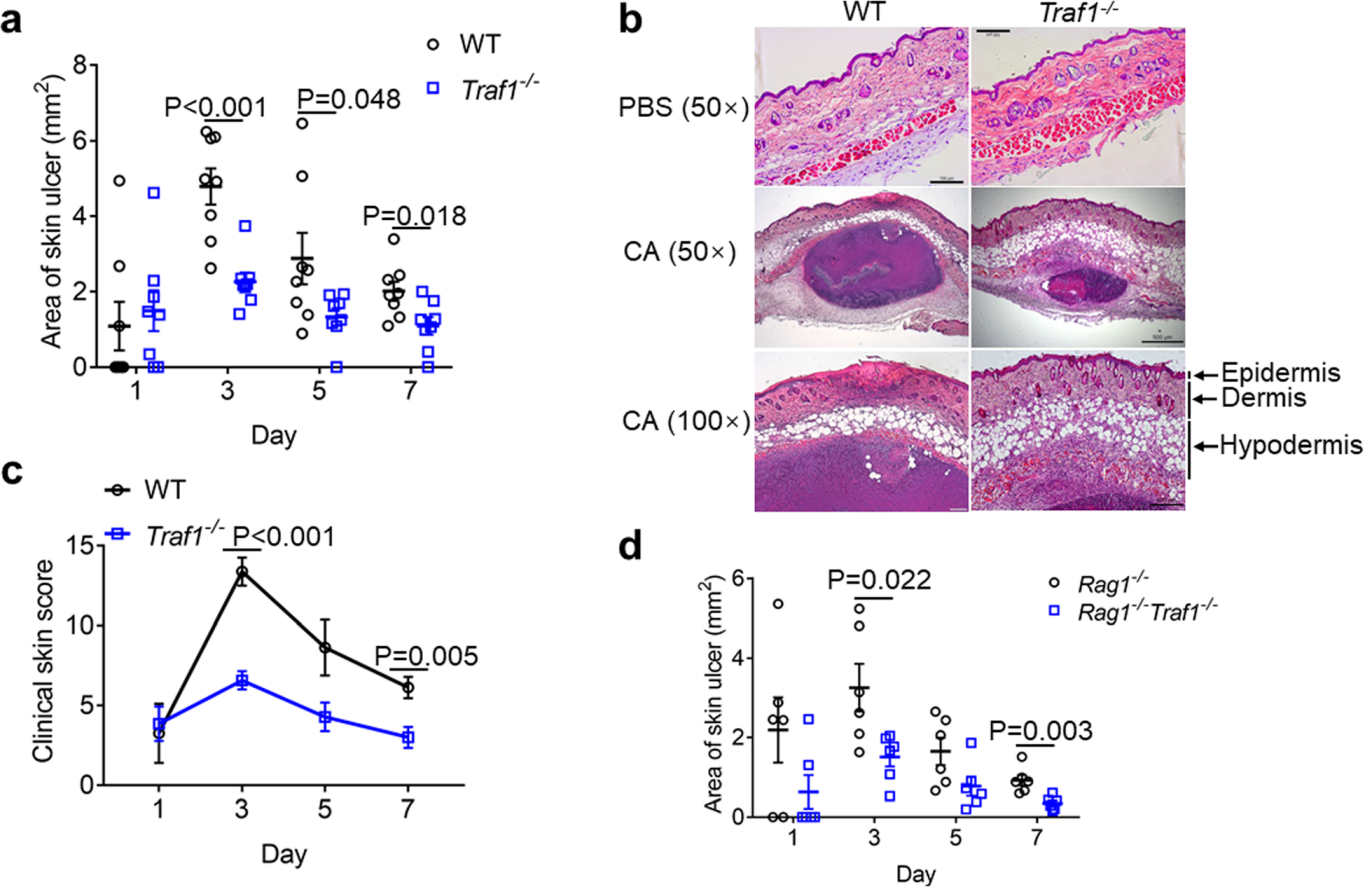
TRAF1-deficiency protects mice from C. albicans intradermal infection**. a**. T*raf1*^*−/−*^ mice (*n* = 8) and WT controls (n = 8) were intradermally infected with *C. albicans* SC5314 (1 × 10^7^ CFU) and the area of the skin ulcer was measured every other day. **b**. Paraffin-embedded skin sections from WT and *Traf1*^*−/−*^ mice 3 days post-infection were stained by H&E. Representative micrographs were captured at 50× and 100× magnification. Arrows indicate different layers of the skin region. PBS group, the control group; CA group, the *C. albicans* infection group. **c**. *Traf1*^*−/−*^ mice (n = 8) and WT controls (n = 8) were intradermally infected with *C. albicans* (1 × 10^7^ CFU). The clinical scores (scale 0–16) were the sum of individual scores graded as 0 (none), 1 (slight), 2 (moderate), 3 (marked) and 4 (very marked) for ulceration, scab, erythema and nodule, which were recorded every other day for a total of 7 days. The observer was blinded to all biopsy specimens. **d**. *Rag1*^*−/−*^*Traf1*^*−/−*^ mice (*n* = 6) and *Rag1*^*−/−*^ controls (n = 6) were intradermally infected with *C. albicans* (1 × 10^7^ CFU), and the skin ulcer was measured every other day for a total of 7 days. Individual points represent different mice. Data are pooled from two independent experiments and shown as mean ± SEM, and were analyzed using the unpaired, two-tailed, Student’s t-test. Values of p below 0.05 represented a statistically significant difference

Although TRAF1 has been implicated in the control of rheumatoid arthritis through its regulation on LPS-induced proinflammatory response [19], whether TRAF1 plays a role in *Candida albicans* infection is unknown. To test this, we intradermally infected TRAF1-deficient mice and assessed the pathology in the skin. TRAF1-deficient mice had a smaller area of ulcer after infection for 3, 5, or 7 days compared to WT mice (Fig. 1a). We found that hypodermal areas enlarged after *C. albicans* infection in both TRAF1-deficient and WT mice, whereas TRAF1-deficient mice exhibited a significant increase in lymphocyte infiltration to the hypodermal layer after *C. albicans* infection for 3 days (Fig. 1b). By assessing the extent of ulceration, scab, erythema, and nodule, we found that TRAF1-deficient mice had less severe clinical symptoms after infection for 3 and 7 days, compared to WT controls (Fig. 1c). Since TRAF1 also plays a critical role in B cells and T cells [20, 21], we then used RAG1-deficient mice, which have impaired adaptive immunity [22] to test whether TRAF1’s role in the adaptive immune compartment contributed to anti-fungal defense in the skin. *Rag1*^*−/−*^ mice were bred with *Traf1*^*−/−*^ mice to generate *Rag1*^*−/−*^*Traf1*^*−/−*^ mice. *Rag1*^*−/−*^*Traf1*^*−/−*^ mice had a smaller area of ulcer after infection for 3 and 7 days, compared to *Rag1*^*−/−*^ mice (Fig. 1d), suggesting that TRAF1’s role in the innate immune cells is pivotal for the immune defense against *C. albicans* intradermal infection. Taken together, TRAF1 has a crucial role in the regulation of innate immune defense against *C. albicans* intradermal infection.

### TRAF1-regulated immune response controls fungal burden

**Fig. 2:**
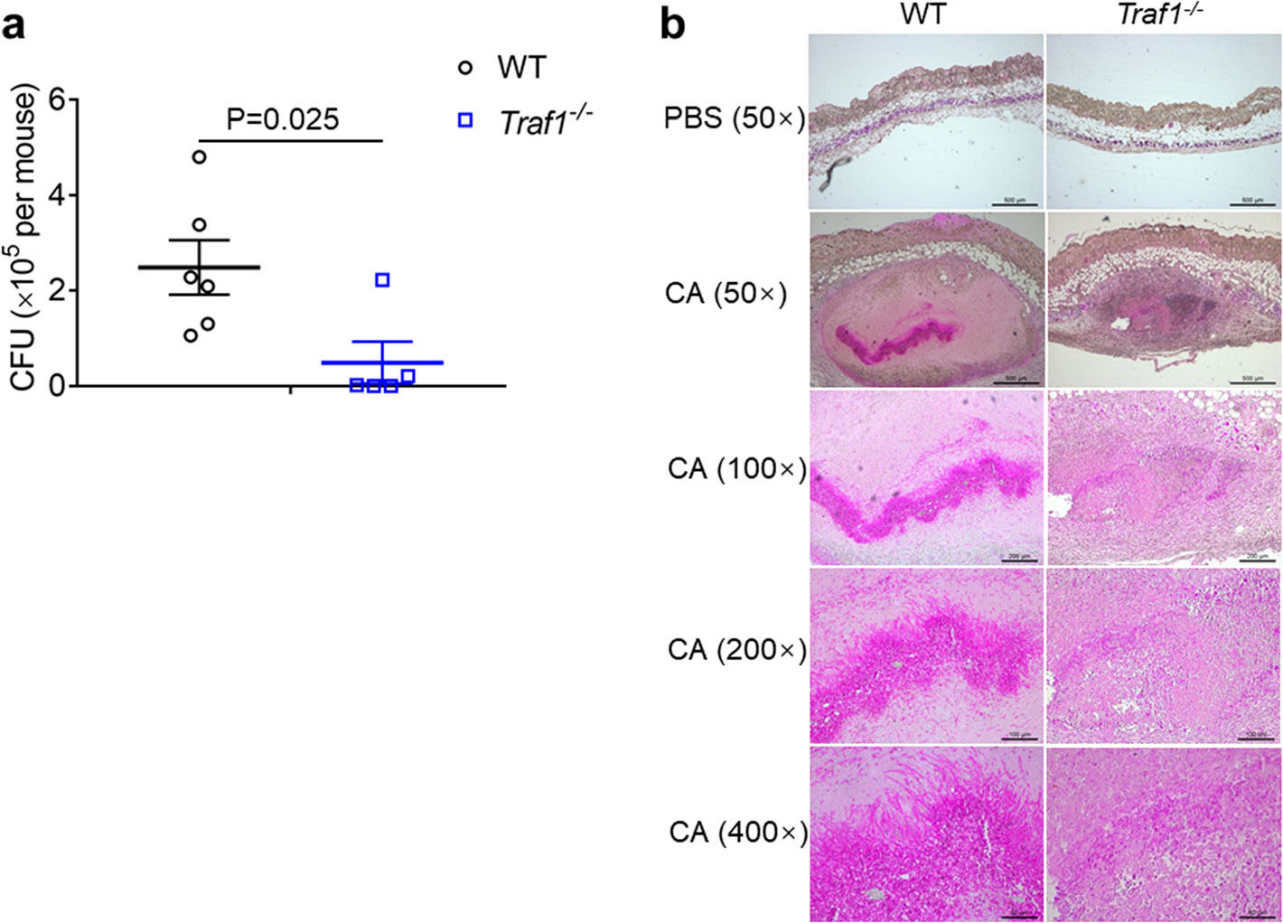
TRAF1-deficiency accelerates the clearance of *C. albicans***. a**. *Traf1*^*−/−*^ mice (*n* = 5) and WT controls (n = 6) were infected with *C. albicans* (1 × 10^7^ CFU) and analyzed for fungal growth within the skin tissue on day 3 post-infection. **b**. Paraffin-embedded skin sections from *Traf1*^*−/−*^ and WT mice 3 days post-infection were stained by PAS. Representative micrographs were captured at 50×, 100×, 200× and 400× magnification. PBS group, the control group; CA group, the *C. albicans* infection group. Data are pooled from two independent experiments and shown as mean ± SEM, and were analyzed using the unpaired, two-tailed, Student’s t-test. Values of p below 0.05 represented a statistically significant difference

Innate immunity contributes to fungal clearance during mucosal *C. albicans* infection [23]. Consequently, we determined the impact of TRAF1 on fungal clearance at the early stage of *C. albicans* infection. We intradermally infected WT and TRAF1-deficient mice with *C. albicans* and then assessed fungal burden in the skin, through CFU counting and PAS staining. TRAF1-deficiency led to a significant reduction in fungal burden after infection for 3 days (Fig. 2a, b), indicating a critical role for TRAF1-regulated immune response in the eradication of invaded *C. albicans*.

### TRAF1 regulates neutrophil recruitment to contain fungal pathogens

**Fig. 3:**
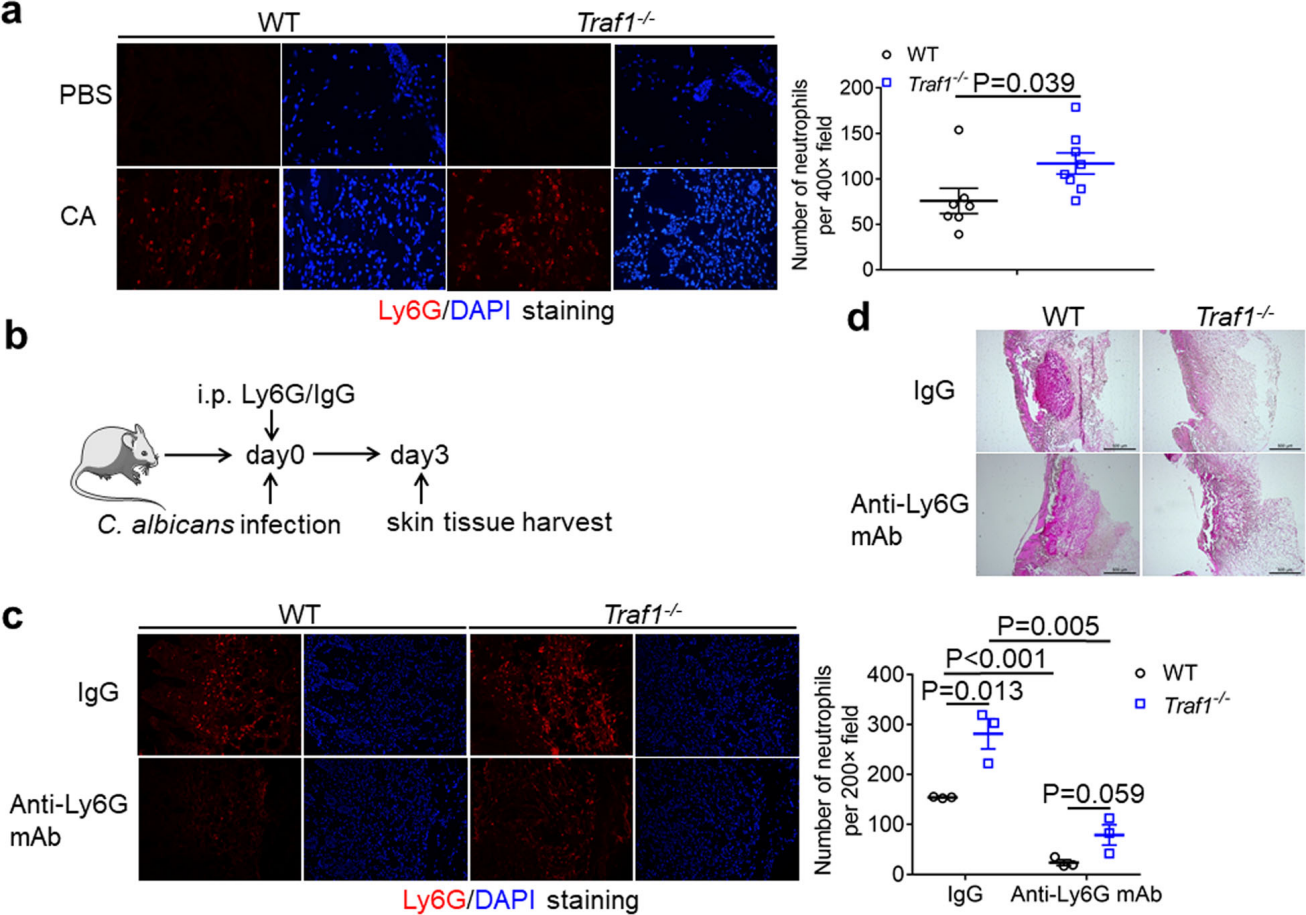
TRAF1-deficiency promotes neutrophil recruitment**. a**. OCT-embedded back skin sections from *Traf1*^*−/−*^ and WT mice infected with *C. albicans* (1 × 10^7^ CFU) were stained by Ly6G antibody (red) and DAPI (blue). The Ly6G^+^ Cells in the skin tissues from *Traf1*^*−/−*^ (n = 8) and WT mice (*n* = 7) were quantified by the average of positive cells from 5 objective fields (400×) each mouse. PBS group, the control group; CA group, the *C. albicans* infection group. **b**. *Traf1*^*−/−*^ mice (*n* = 3) and WT mice (n = 3) were treated with 40 μg of Ly6G mAb or control IgG via intraperitoneal injection (i.p.) 2 h before *C. albicans* (1 × 10^7^ CFU) infection. The Skin tissues were harvested on day 3 after infection for immunofluorescence and PAS staining. **c**. OCT-embedded back skin sections from *Traf1*^*−/−*^ and WT mice pretreated wtih Ly6G mAb or IgG were stained by Ly6G antibody (red) and DAPI (blue), and the Ly6G^+^ cells in the skin tissues were quantified by an average of positive cells from 3 objective fields (200×) each mouse. **d**. OCT-embedded skin sections from *Traf1*^*−/−*^ mice and WT mice pretreated with Ly6G mAb or IgG were stained by PAS. Representative micrographs were captured at 50× magnification. Data are shown as mean ± SEM, and were analyzed using the unpaired, two-tailed, Student’s t-test. Values of p below 0.05 represented a statistically significant difference

Neutrophils are the first responding leukocytes recruited in large numbers to the infected site to clear fungi [24–26]. To determine the neutrophils recruitment, we assessed neutrophils by immunofluorescence staining after *C. albicans* challenge for 3 days. Compared to WT mice, TRAF1-deficient mice had more neutrophils in their skin tissue after infection (Fig. 3a). To determine the role of neutrophils in fungal clearance, we depleted neutrophils in TRAF1-defecient mice with Ly6G antibody via intraperitoneal injection (Fig. 3b). After *C. albicans* challenge for 3 days, neutrophil recruitment was evident in both WT and TRAF1-deficient skins treated with IgG antibody, and TRAF1-deficient mice had more neutrophil infiltration than WT mice (Fig. 3c).

**Fig. 4:**
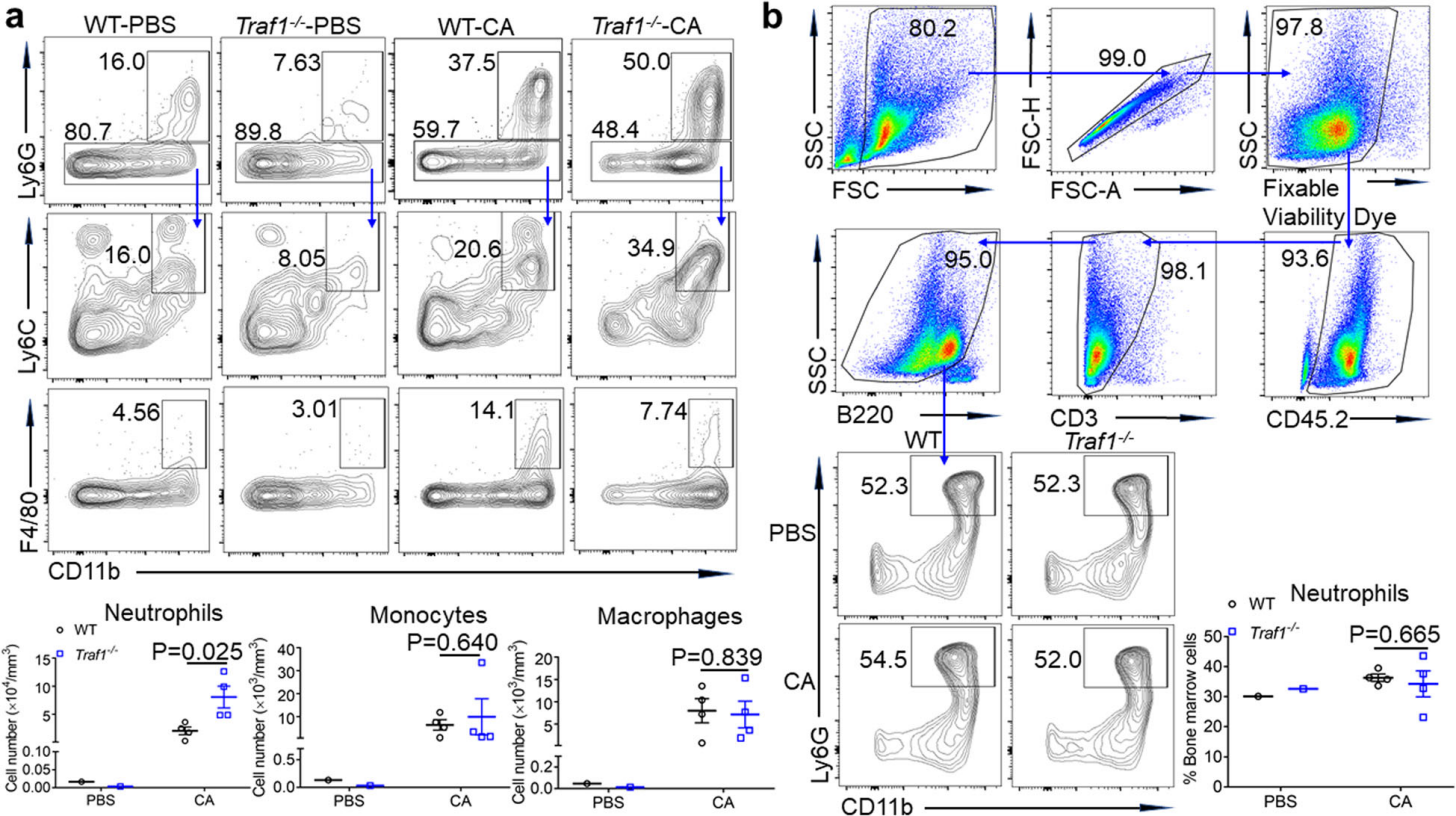
TRAF1-deficiency promotes the recruitment of neutrophils but not granulopoiesis. *Traf1*^*−/−*^ mice (*n* = 4) and WT controls (n = 4) were infected with *C. albicans* (1 × 10^7^ CFU) or PBS for 3 days. **a**. Skin tissue single-cell suspensions were analyzed by flow cytometry. Representative plots were gated on neutrophils (top: CD45^+^CD11b^+^Ly6G^+^), monocytes (middle: CD45^+^Ly6G^−^CD11b^+^Ly6C^+^) and macrophages (bottom: CD45^+^CD11b^+^F4/80^+^). Cell number were calculated: the percentage of skin single cell suspensions × the number of skin single cell suspensions/the skin volume (mm^3^). **b**. Bone marrow cell suspensions were analyzed by flow cytometry. Bone marrow single live cell suspensions were Fixable Viability Dye negative. Representative plots were gated on neutrophils (CD45^+^CD3^−^B220^−^CD11b^+^Ly6G^+^). The percentages were calculated as percentages of total bone marrow cells. PBS group, the control group; CA group, the *c. albicans* infection group. Data are shown as mean ± SEM and were analyzed using the unpaired, two-tailed, Student’s t-test. Values of p below 0.05 represented a statistically significant difference. Values of p below 0.05 represented a statistically significant difference

In contrast, following Ly6G antibody treatment, both WT and TRAF1-deficient mice had reduced neutrophil infiltration, which became comparable between WT and TRAF1-deficient mice (Fig. 3c). Meanwhile, while IgG-treated TRAF1-deficient mice showed a significant reduction in fungal burden than the WT controls after infection for 3 days, TRAF1-deficient mice treated with Ly6G antibody exhibited uncontrolled fungal growth 3 days post-infection, just like the control WT mice (Fig. 3d). Therefore, TRAF1-regulated *C. albicans* clearance in skin tissue is dependent on neutrophil recruitment. To further assess the recruitment of neutrophils to the fungi-infected skin, we also performed flow cytometry. After challenge with *C. albicans* for 3 days, TRAF1-deficient skin had more infiltrated neutrophils than WT controls, whereas the infiltrated monocytes and macrophages were comparable in both genotypes (Fig. 4a). By examining the neutrophils in the bone marrow, we found that both WT and TRAF1-deficient mice had similar numbers of neutrophils in their bone marrows before or after *C. albicans* infection (Fig. 4b), hence ruling out a possible role for TRAF1 in granulopoiesis. Taken together, TRAF1 plays a critical role in the regulation of neutrophil recruitment for *C. albicans* clearance.

### TRAF1 regulates chemokine CXCL1 production in *C. albicans* infection

**Fig. 5:**
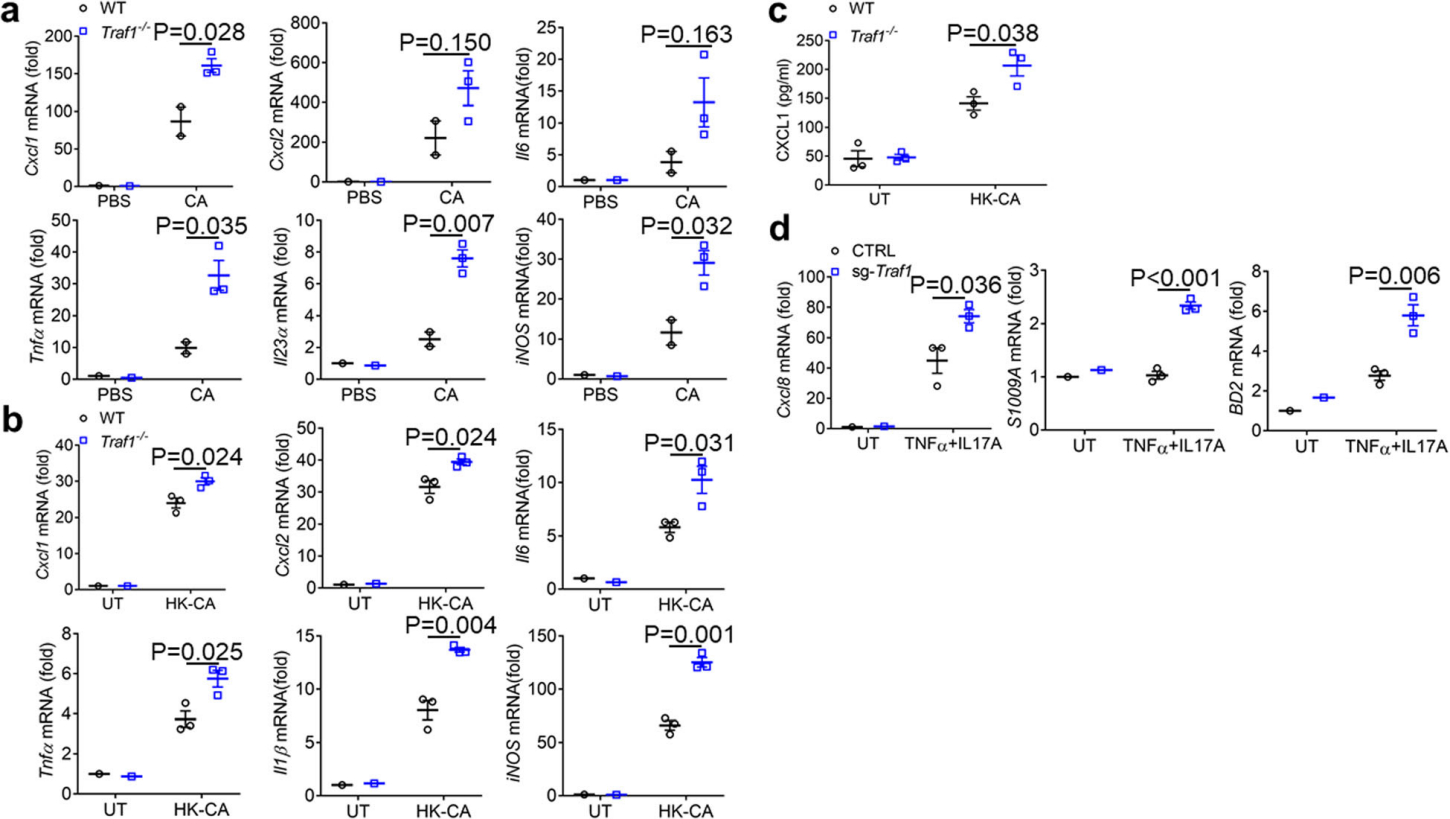
TRAF1-deficiency promotes CXCL1 production with *C. albicans* infection. **a**. *Traf1*^*−/−*^ mice (n = 3) and WT controls (*n* = 2) were infected with *C. albicans* (1 × 10^7^ CFU) or PBS and analyzed for gene expression within the skin tissue 3 days post-infection by qPCR. Results were reported as fold change normalized to the expression of β-actin and relative to PBS control. PBS group, the control group; CA group, the *c. albicans* infection group. **b**. *Traf1*^*−/−*^ and WT BMDMs pretreated with IL-4 (10 ng/ml) O/N were unstimulated or stimulated with HK-CA (MOI = 5) for 6 h and the expression of *Cxcl1, Cxcl2, Il6, Ilβ, Tnfα, iNOS* was evaluated by qPCR. Results were reported as fold change normalized to the expression of β-actin and relative to untreated control. **c**. *Traf1*^*−/−*^ and WT BMDMs pretreated with IL-4 (10 ng/ml) O/N were stimulated with HK-CA (MOI = 5) or not for 24 h and the protein levels of CXCL1 were evaluated by ELISA. **d**. CTRL and sg-*Traf1* HaCaT cells were either stimulated or unstimulated with TNFα (20 μg/ml) plus IL17A (50 μg/ml) for 9 h, and the expression of *Cxcl8, S100A9, BD2* was evaluated by qPCR. Results were reported as fold change normalized to the expression of β-actin and relative to untreated control. Data are shown as mean ± SEM and are representative of at least two experiments performed in triplicate. Data were analyzed using the unpaired, two-tailed, Student’s t-test. Values of p below 0.05 represented a statistically significant difference

Neutrophils are recruited to the infection site by chemokines such as CXCL1 and CXCL2 [27]. To determine the local cues responsible for the recruitment of neutrophils to the infected skin area, we examined the induction of a subset of chemokines and cytokines during *C. albicans* intradermal infection. TRAF1-deficient mice had more abundant CXCL1 and proinflammatory cytokines such as TNFα and IL-23 in the *C. albicans*-infected skin tissues (Fig. 5a), suggesting that TRAF1 controls CXCL1 production. Next, we detected CXCL1 and cytokine expression in macrophages with heat-killed *C. albicans* stimulation. In line with our work in vivo, TRAF1-deficient macrophages had increased mRNA and protein levels of CXCL1 (Fig. 5b, c) and higher amounts of cytokines such as TNFα and IL-1β (Fig. 5b). Consistent with a synergy of TNF and IL-17 in the induction of CXCL8, the human homolog of CXCL1 [28], TRAF1-deficient keratinocytes also elevated CXCL8 expression following TNF + IL-17 stimulation (Fig. 5d). Thus, our data implicate a critical role for TRAF1 in the regulation of CXCL1 expression in both macrophages and keratinocytes.

### CXCL1 is responsible for the elevation of neutrophil infiltration in TRAF1-deficient mice

**Fig. 6:**
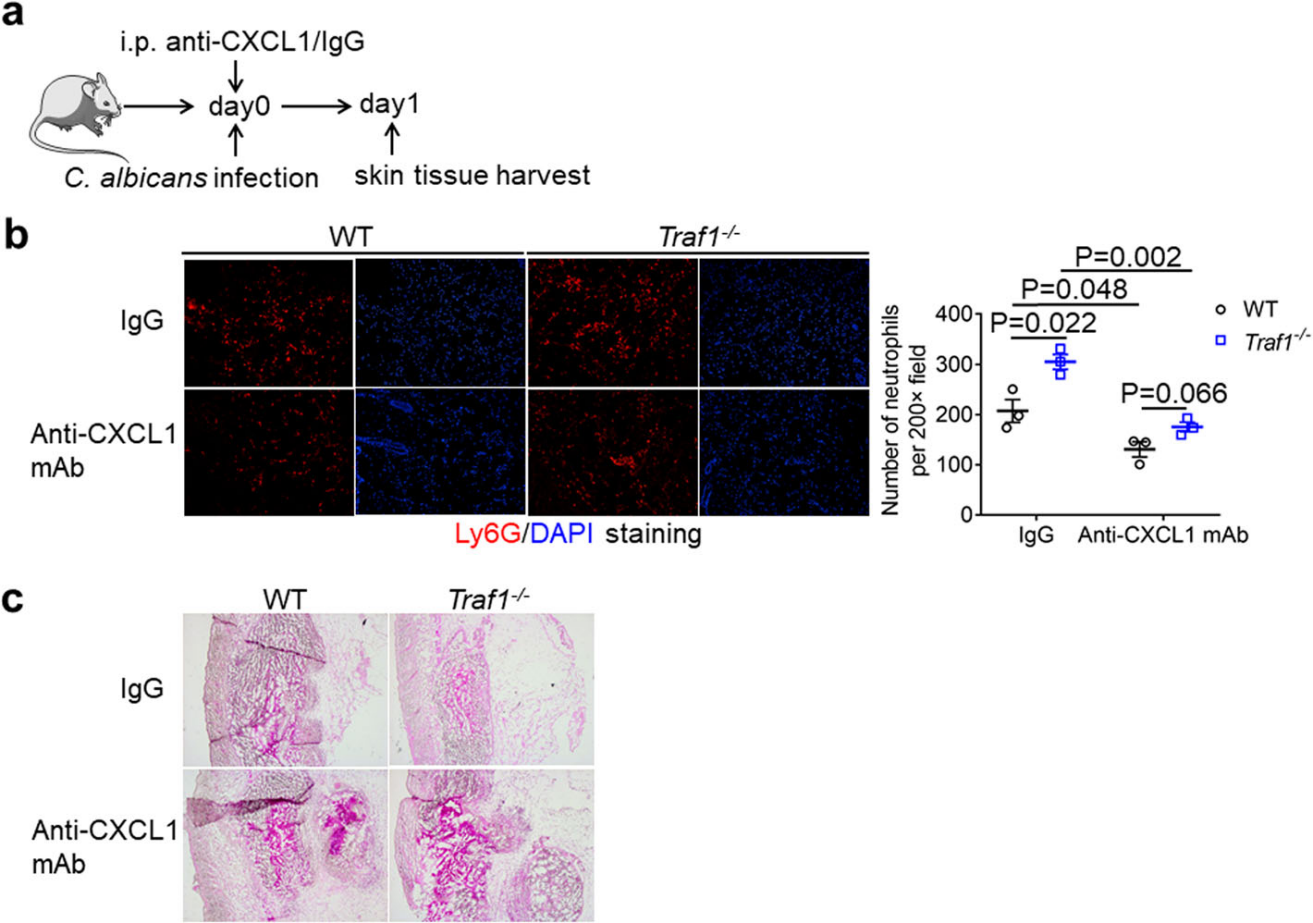
CXCL1 is responsible for the elevation of neutrophil infiltration in TRAF1-deficient mice. **a**. *Traf1*^*−/−*^ mice (n = 3) and WT mice (n = 3) were treated with 40 μg of anti-CXCL1 mAb or control IgG via intraperitoneal injection 2 h prior to *C. albicans* (1 × 10^7^ CFU) infection. The Skin tissue was harvested one day after infection for immunofluorescence staining and PAS staining. **b**. OCT-embedded back skin sections from *Traf1*^*−/−*^ and WT mice pretreated with anti-CXCL1 mAb or IgG were stained by Ly6G antibody (red) and DAPI (blue), and the Ly6G^+^ cells were quantified by an average of positive cells from 3 objective fields (200×) each mouse. **c**. OCT-embedded skin sections from *Traf1*^*−/−*^ and WT mice treated with anti-CXCL1 mAb or IgG were stained by PAS. Representative micrographs were captured at 50× magnification. Data are shown as mean ± SEM, and were analyzed using the unpaired, two-tailed, Student’s t-test. Values of p below 0.05 represented a statistically significant difference

Subsequently, we neutralized CXCL1 with anti-CXCL1 antibody, thereby investigated its role in neutrophil infiltration in TRAF1-deficient mice (Fig. 6a). After *C. albicans* challenge for one day, TRAF1-deficient mice pretreated with control IgG antibody showed increased neutrophil recruitment in the skin, whereas TRAF1-deficient mice with anti-CXCL1 pretreatment had severely impaired neutrophil recruitment (Fig. 6b). In contrast to IgG-treated mice, no significant difference in neutrophil recruitment was observed between WT and TRAF1-deficient mice with anti-CXCL1 antibody treatment (Fig. 6b). Further, while TRAF1-deficiency led to a significant reduction in fungal burden after *C. albicans* infection for one day, TRAF1-deficient mice treated with anti-CXCL1 antibody exhibited uncontrolled fungal growth (Fig. 6c), suggesting a central role for CXCL1 in TRAF1-regulated neutrophil infiltration and fungal clearance.

### TRAF1 regulates NFκB or STAT1 activation in macrophages or keratinocytes, respectively

**Fig. 7:**
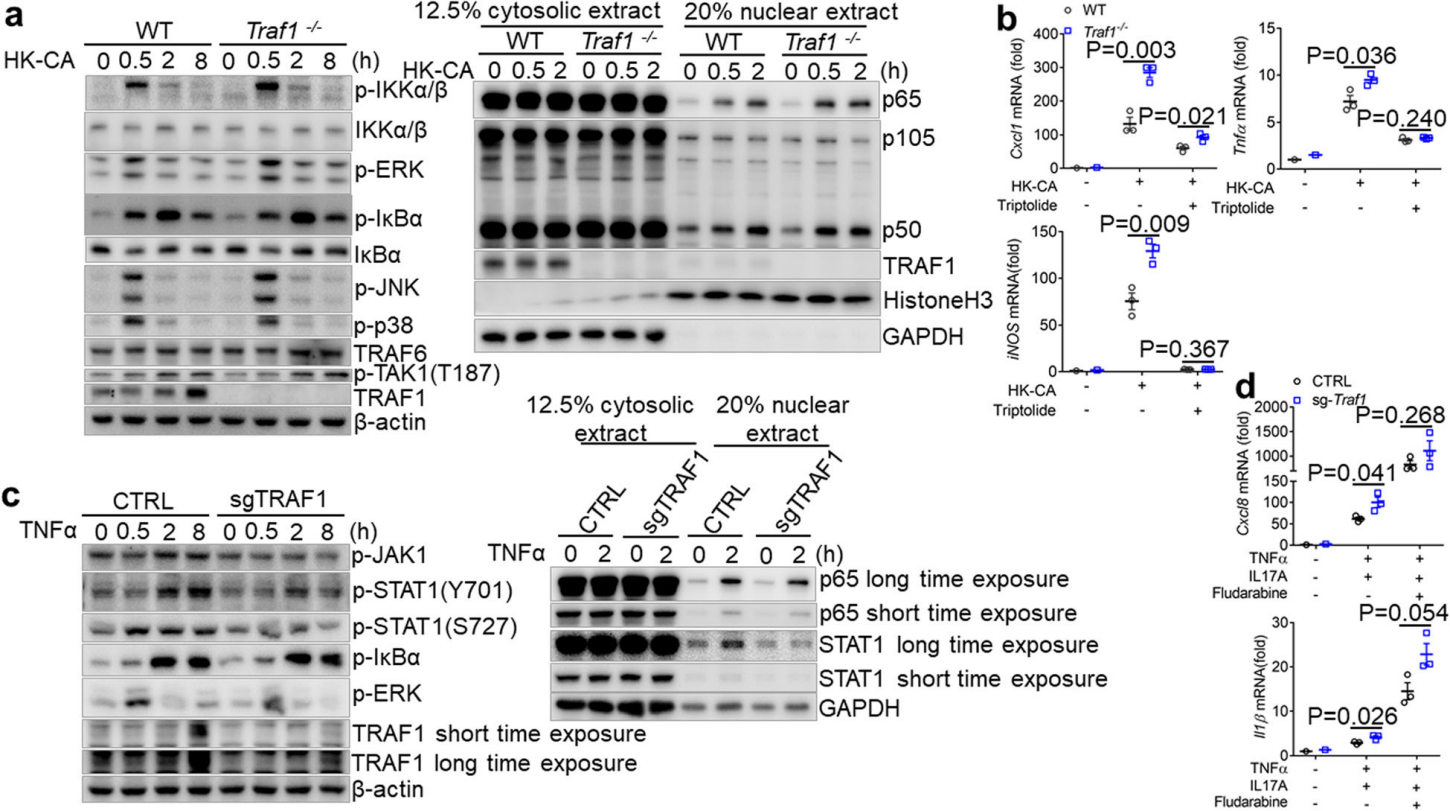
TRAF1-deficiency influences p65 in macrophages and STAT1 in keratinocytes. **a**. *Traf1*^*−/−*^ and WT BMDMs pretreated with IL-4 (10 ng/ml) O/N were treated with HK-CA (MOI = 5) for indicated times. The whole cell lysates or cytosolic/nuclear extracts were prepared and probed with indicated antibodies. **b**. IL-4 (10 ng/ml) pretreated *Traf1*^*−/−*^ and WT BMDMs were pretreated with triptolide (20 ng/ml) for 2 h before HK-CA (MOI = 5) treatment for 6 h. The expression of *Cxcl1, iNOS, Tnfα* was evaluated by qPCR. Results were reported as fold change normalized to the expression of β-actin and relative to untreated control. **c**. CTRL and sg-*Traf1* HaCaT cells were stimulated by TNFα (20 μg/ml) for indicated times. The whole cell lysates or cytosolic/nuclear extracts were prepared and probed with indicated antibodies. **d**. CTRL and sg-*Traf1* HaCaT cells were either stimulated or unstimulated with TNFα (20 μg/ml) plus IL17A (50 μg/ml) for 9 h, in the absence or presence of fludarabine (100 μM) for 4 h, and the expression of *Cxcl8, Ilβ* was evaluated by qPCR. Results were reported as fold change normalized to the expression of β-actin and relative to untreated control. For Q-PCR, data are shown as mean ± SEM and are representative of at least two experiments performed in triplicate, and were analyzed using the unpaired, two-tailed, Student’s t-test. Values of p below 0.05 represented a statistically significant difference. The Western blots are representative of at least three independent experiments

Next, we investigated the signaling pathway activated by heat-killed *C. albicans* in macrophages. Following stimulation with heat-killed *C. albicans*, we found that while the phosphorylation of IκBα was unaltered between WT and TRAF1-deficient macrophages, the nuclear translocation of NFκB p65 and p50 was increased in TRAF1-deficient macrophages (Fig. 7a), indicating elevated NFκB activation. Since NF-κB signaling has been previously implicated in the induction of CXCL1 in the macrophages [29], we subsequently detected CXCL1 expression in these cells. Upon stimulation with heat-killed *C. albicans*, TRAF1-deficient macrophages expressed much higher *Cxcl1* than WT controls (Fig. 7b). Moreover, the induction of proinflammatory mediators such as *Tnfα* and *iNos* was also upregulated in TRAF1-deficient macrophages (Fig. 7b). Notably, pretreatment with NFκB inhibitor triptolide led to decreased *Cxcl1* expression, which became comparable in both WT and TRAF1-deficient macrophages (Fig. 7b), suggesting TRAF1 may temper NFκB activation in the control of *Cxcl1* expression in macrophages.

Interestingly, while TRAF1-deficient keratinocytes exhibited similar levels of IκBα phosphorylation to WT controls, they had impaired phosphorylation of STAT1 in response to TNF stimulation (Fig. 7c). Consistently, the nuclear translocation of STAT1 was also markedly reduced in TRAF1-deficient keratinocytes (Fig. 7c). Given STAT1 has been reported to be able to negatively regulate LPS-induced proinflammatory gene expression [30]; hence we examined the role of STAT1 in TRAF1-induced CXCL8 expression. To that end, we detected CXCL8 expression in both WT and TRAF1-deficient keratinocytes stimulated by TNFα plus IL-17 (to stabilize CXCL1 mRNA [31]) with pretreatment of fludarabine, an inhibitor of STAT1. TRAF1-deficient keratinocytes had increased expression of CXCL8 and IL-1β than WT controls, and fludarabine treatment led to more abundant CXCL8 expression in both WT and TRAF1-deficient keratinocytes (Fig. 7d). However, the significant difference between WT and TRAF1-deficient keratinocytes in CXCL8 expression disappeared upon fludarabine treatment (Fig. 7d), which suggests TRAF1 may regulate CXCL8 expression through controlling STAT1 activation in the keratinocytes. Taken together, TRAF1 may be able to regulate CXCL1 expression in both macrophages and keratinocytes, via the control of NFκB or STAT1 activation, respectively.

### TRAF1-deficiency in the macrophages plays a critical role in anti-fungal defense

**Fig. 8:**
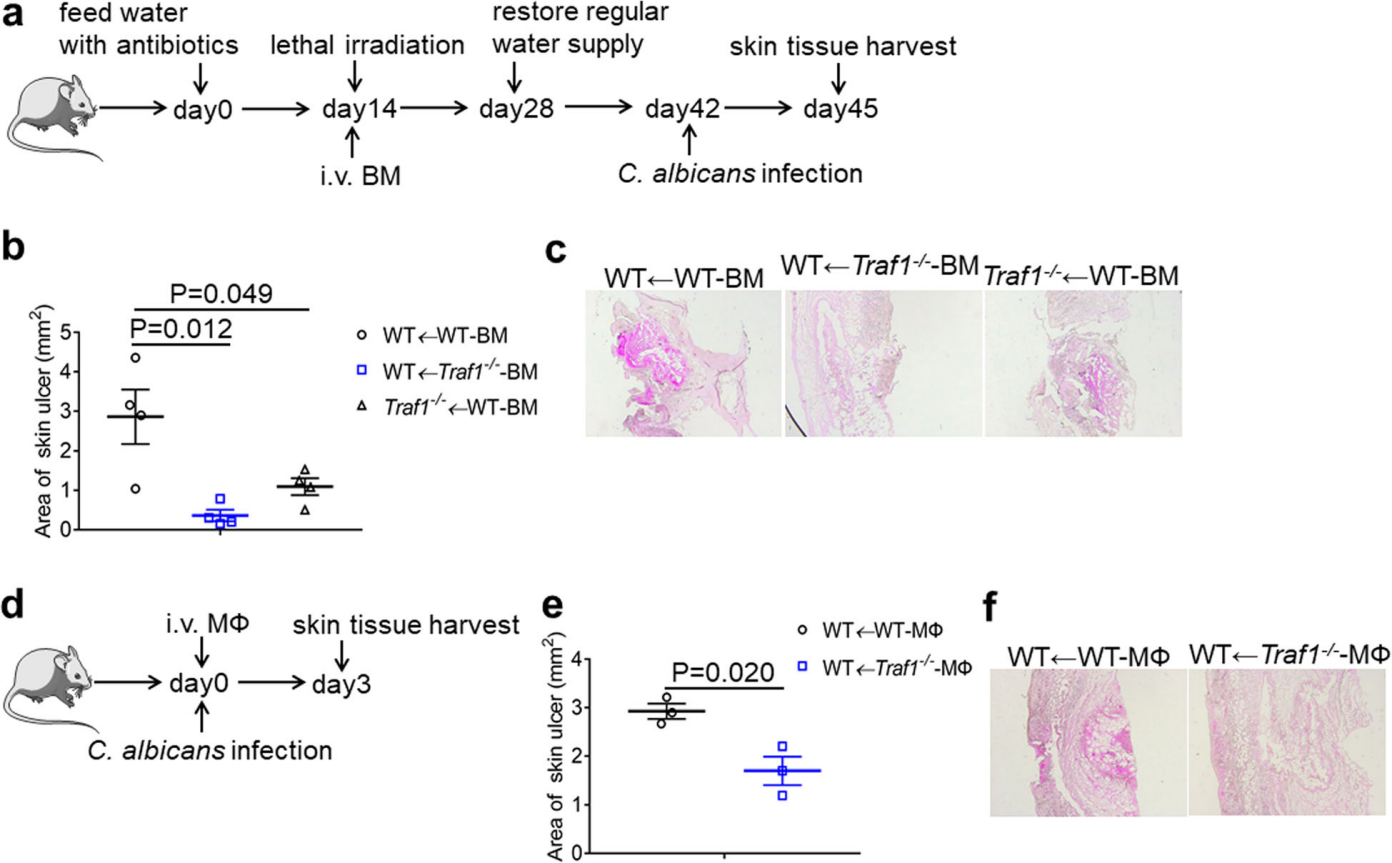
TRAF1-deficient macrophages play a critical role in *C. albicans* skin infection in vivo. **a**. WT mice (*n* = 8, 6 weeks old) and *Traf1*^*−/−*^ (n = 4, 6 weeks old) recipient mice were fed with antibiotics in the drinking water for 2 weeks and were lethally irradiated. In 4 h, bone marrow cells (BM) from WT or *Traf1*^*−/−*^ donors were injected intravenously (i.v.) (1 × 10^6^ cells /mouse) into the recipient mice. Antibiotics in drinking water were fed for another 2 weeks and then regular water was restored for another 2 weeks before challenged with *C. albicans* for 3 days. **b**. The area of the skin ulcer of reconstituted mice 3 days post-infection was measured. **c**. OCT-embedded skin sections from reconstituted mice 3 days post-infection were stained by PAS. Representative micrographs were captured at 50× magnification. **d**. WT and *Traf1*^*−/−*^ macrophages (MФ) were injected intravenously (1 × 10^6^ cells /mouse) into WT mice. In 4 h, recipient mice were challenged with *C. albicans* for 3 days. **e**. The area of the skin ulcer of recipient mice 3 days post-infection was measured. **f**. OCT-embedded skin sections from recipient mice 3 days post-infection were stained by PAS. Representative micrographs were captured at 50× magnification. Data are shown as mean ± SEM, and were analyzed using the unpaired, two-tailed, Student’s t-test. Values of p below 0.05 represented a statistically significant difference

As TRAF1-deficiency was able to boost CXCL1 production in both macrophages and keratinocytes, we next generated bone marrow chimeric mice to address the cell type-specific role for TRAF1 in fungal infection. By transferring WT or TRAF1-deficient bone marrow cells to lethal irradiated WT or TRAF1-deficient recipients reciprocally, we generated three type of chimeras (Fig. 8a). After *C. albicans* challenge for 3 days, WT recipients reconstituted with TRAF1-deficient bone marrows had significantly ameliorated ulcer and fungal burden than WT recipients reconstituted with WT bone marrows (Fig. 8b, c). These data thus suggest a critical role for the hematopoietic compart-derived TRAF1 in the regulation of anti-fungal immune defense. On the other hand, TRAF1-deficient recipients reconstituted with WT bone marrow cells also had a smaller area of ulcer and less fungal burden compared to WT mice reconstituted with WT bone marrow cells (Fig. 8b, c), hence suggesting that the stromal TRAF1 also played a significant role in mounting the immune defense against fungal skin infection. Together, these data demonstrate that TRAF1 expression in both innate immune compartment and keratinocytes contribute to the regulation of anti-fungal immune defense.

**Fig. 9:**
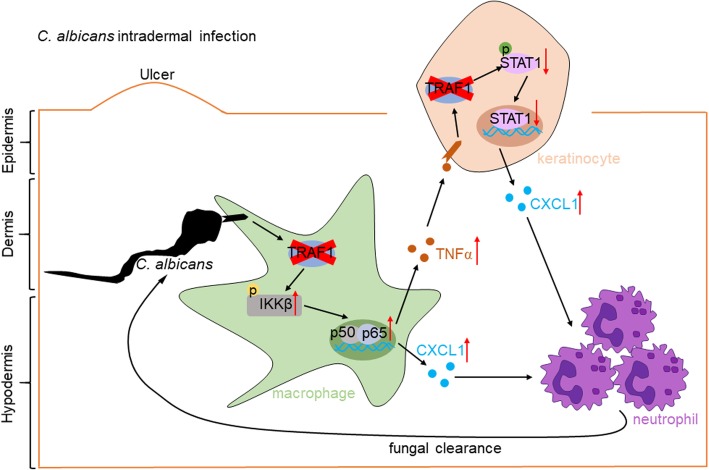
Model of TRAF1-mediated antifungal immunity in the skin. Schematic presentation of the role of TRAF1 during *C. albicans* intradermal infection. TRAF1-deficiency upregulates CXCL1 production and neutrophil recruitment through increasing the activation of NFκB in the macrophages or decreasing STAT1 activation in the keratinocytes, respectively, leading to a better clearance of fungi in the skin

To further analyze the role of TRAF1-deficiency in macrophages in vivo, we adoptively transferred TRAF1-deficient macrophages to WT mice and infected them with *C. albican*s (Fig. 8d). After *C. albicans* challenge for 3 days, WT mice transferred with TRAF1-deficient macrophages had smaller area of ulcer and less fungal pathogen compared to WT mice transferred with WT macrophages (Fig. 8e, f), suggesting that TRAF1-deficient macrophages play a critical role in protecting against *C. albicans* skin infection in vivo. Together, we demonstrate that TRAF1 plays a crucial role in the regulation of the immune defense against the intradermal infection with *Candida albicans*, by controlling the activation of NFκB and STAT1 in the macrophages or keratinocytes, respectively, and reveal a central role for CXCL1-neutrophil axis in the containing of fungal infection in the skin (Fig. 9).

## Discussion

In the present study, we demonstrated a critical contribution of TRAF1-mediated CXCL1 expression in the host defense against *C. albicans* infection in the skin tissue. We demonstrated that TRAF1 negatively regulates CXCL-1 production likely through the control of NFκB and STAT1 activation in both macrophages and keratinocytes. Our results suggest TRAF1-regulated CXCL1 production has a crucial role in the recruitment of neutrophil and the eradication of invaded fungal pathogens. Collectively, our study identifies a critical role for TRAF1 in fungal infection and offers new insights into the host defense mechanisms in containing *C. albicans* intradermal infection.

*C. albicans* is the most common human fungal pathogen, which naturally colonizes the skin, genital, and intestinal mucosa of healthy individuals [32, 33]. Pattern recognition of *C. albicans* via TLRs and CLRs leads to the induction of Th1 and Th17 response, which promotes the recruitment and activation of neutrophil and macrophages in killing *C. albicans* [9, 34]. Neutropenia is the major predisposing factor for systemic candidiasis [35]. CARD9-deficiency causes *C. albicans* CNS disease by a brain-specific defect in neutrophil recruitment [25]. The defect in IL-17 production renders patients highly susceptible to CMC [36]. Therefore, the inherited immune deficiency is one of the predominant factors contributing to *C. albicans* infection. Although a TRAF1 polymorphism has been linked to rheumatoid arthritis [37], the role of TRAF1 in *C. albicans* infection was previously unknown. Our study firstly revealed that TRAF1-deficiency can exert a protective role in *C. albicans* intradermal infection, thus highlighting the complex regulation of immune responses through TRAF1.

The adaptive immunity is critical for the control of *C. albicans* infection [38]. For example, the Th1 cell response is critical for the host defense against the systemic infection with *C. albicans*, whereas the Th17 cell response has an indispensable role in the immune protection against *C. albicans* cutaneous infection [39, 40]. CD4^+^ IL-17-producing TRM cells can mediate long-term protective immunity against *C. albicans* skin infection [41]. Although TRAF1 is well-known for its role in the regulation of B cell and T cell functions [20, 21], we found that *Rag1*^*−/−*^*Traf1*^*−/−*^ mice were still able to mount a better immune defense against fungal infection than *Rag1*^*−/−*^ mice. Hence, TRAF1’s expression and function in the lymphocytes seem dispensable in the regulation of anti-fungal skin immune response. Besides, we also generated bone marrow chimeric mice, thereby demonstrated that both innate immune cells such as neutrophils and macrophages and tissue cells such as keratinocytes might have contributed to TRAF1-regulated immune protection against *C. albicans* infection. Therefore, our results reveal a complex interaction and crosstalk between immune cells and non-immune cells in the induction of concerted host defense against fungal infection in the skin.

CXCL1 and its human homolog CXCL8 can be secreted by a variety of cell types, including alveolar macrophages, epidermal cells, blood monocytes, fibroblasts and endothelial cells [42]. In our study, we focused on CXCL1/CXCL8 expression in macrophages and keratinocytes, respectively. CXCL1/CXCL8 can be induced by cytokines such as TNF and IL-1, as well as bacterial component LPS and fungal-components β-glucan and mannan [43]. The induction of CXCL1/8 involves two major mechanisms: transcriptional initiation through NF-κB and AP-1, and mRNA stabilization through p38MAPK [44]. IKK complex composed of IKKα/IKKβ and NEMO can directly phosphorylate IκBα, leading to its degradation and the release and nuclear translocation of NF-κB p65/p50 heterodimers [45]. Our results showed that TRAF1-deficiency enhanced the phosphorylation of IKKβ and nuclear translocation of p65 in macrophages. This mechanism might have contributed to the upregulation of CXCL-1 expression in the macrophages as blockade of p65 with triptolide abrogated this effect. One the other hand, STAT1 can inhibit NF-κB activity through its interaction with AhR, thereby suppressing LPS-induced response [30] or downregulating TNFα-mediated NF-kB activation through TNFR1 and TRADD [46]. Thus these studies suggest that STAT1 can act as a negative regulator of NF-κB in certain circumstances. Our data demonstrate that TRAF1-deficiency impairs the phosphorylation and nuclear translocation of STAT1 in keratinocytes, a phenomenon that may underlie the upregulation of CXCL8 in these cells. Thus, our study implicates a novel mechanism by which TRAF1 regulates NFκB activation through STAT1.

CXCL1 and CXCL8 are the key chemokines recruiting neutrophils to the infected sites under both mucosal and systemic *C. albicans* infection [47, 48]. By blocking CXCL1’s action through neutralizing antibodies, we demonstrate a crucial role for CXCL1 in the recruitment of neutrophils to the infected skin. Neutrophils are the first wave of immune cells migrating to the infected sites, and impaired neutrophil recruitment often leads to uncontrolled propagation of pathogens [49]. Granulocyte transplantation was used to treat invasive fungal infections in patients with neutropenia or neutrophil dysfunction [50]. By employing anti-Ly6G mAb to deplete neutrophils, our results showed that the increased fungal clearance exhibited in TRAF1-deficient mice is contingent on neutrophil recruitment. Although CXCL1 and neutrophils have been previously implicated in *C. albicans* infection, their involvement in the skin infection of *C. albicans* was unclear. In this regard, our study provides new evidence supporting a broad role for the CXCL1-neutrophil axis in the defense against *C. albicans* infection.

## Conclusions

In summary, we firstly demonstrate a crucial role for TRAF1 in the regulation of immune defense against *C. albicans* infection in the skin. Our data also suggests a scenario that TRAF1 may regulates NFκB and STAT1 activation, whereby regulating CXCL1 expression in the macrophages and keratinocytes, respectively. Hence, our work uncovers the complex interaction between immune cells and non-immune cells is imperative of a concerted and effective defense against fungal infection, and TRAF1 has a central role in coordinating this process.

## Data Availability

Not applicable.

## Abbreviations

AMPs: Antimicrobial peptides
AP1: Activator protein-1
BM: Bone marrow cells
BMDM: Bone marrow-derived macrophages
C5: Complement component 5
CARD9: Caspase recruitment domain
CFU: Colony-forming units
CMC: Chronic mucosal and cutaneous candidiasis
CNS: Central nervous system
dDC: Dermal dendritic cells
H&E: Hematoxylin and eosin
HaCaT cells: Immortalized human keratinocytes
HK-CA: Heat-killed *C. albicans*
i.p.: Intraperitoneal injection
i.v.: Intravenous injection
JNK: C-jun n-terminal kinases
L: Length
LPS: Lipopolysaccharide
LUBAC: The Linear Ubiquitin Assembly Complex
MФ: Macrophages
NF-κB: Nuclear factor-kappa B
OCT: Optimum cutting temperature
PAS: Periodic acid schiff
Q-PCR: Real-time PCR
ROS: Reactive oxygen species
TNFR2: Tumor necrosis factor receptor 2
TRAF1: Tumor necrosis factor-associated factor 1
W: Width
